# Supplementary material for: Determinants of contraceptive use among women 0–23 months postpartum in Kitui County, Kenya: A cross-sectional study
Source: PLOS Glob Public Health. 2022 Jun 2;2(6):e0000482. doi: 10.1371/journal.pgph.0000482 (PMC10021444; doi:10.1371/journal.pgph.0000482)
Supplement: Attachment — Submitted filename: Response to reviewers.docx [file pgph.0000482.s001.docx]

**Response to reviewers: Manuscript title “Determinants of Contraceptive Use Among Women 0-23 Months Postpartum in Kitui County, Kenya: A Cross-Sectional Study”**

Ref: PGPH-D-21-00638

We would like to thank the reviewers for careful and thorough reading of this manuscript and for the thoughtful comments and constructive suggestions, which has helped to improve the quality of this manuscript. Our point-by-point responses to the reviewers’ comments follows in the table below. In addition, we have provided as requested, 1) A marked-up copy of your manuscript that highlights changes made to the original version (Revised Manuscript with Track Changes'. 2) An unmarked version of your revised paper without tracked changes (manuscript)

|  | **Comment** | **Feedback/ response to reviewer /journal** |
| --- | --- | --- |
|  | **General comments** | |
| 1 | Please include additional information regarding the survey or questionnaire used in the study and ensure that you have provided sufficient details that others could replicate the analyses. For instance, if you developed a questionnaire as part of this study and it is not under a copyright more restrictive than CC-BY, please include a copy, in both the original language and English, as Supporting Information. | The questionnaire used for the entire baseline evaluation is now provided. The excel file contains tabs for all the other program areas, while the pdf contains the MNH/FP module. The tool was adapted from existing survey questionnaires such as Demographic Health Survey (DHS), Multiple Indicator Cluster Survey (MICS) and Performance Monitoring for Action (PMA). |
| 2 | Please amend your detailed Financial Disclosure statement. This is published with the article, therefore should be completed in full sentences and contain the exact wording you wish to be published.  i). Please include all sources of funding (financial or material support) for your study. List the grants (with grant number) or organizations (with url) that supported your study, including funding received from your institution. ii). State the initials, alongside each funding source, of each author to receive each grant.  iii). State what role the funders took in the study. If the funders had no role in your study, please state: “The funders had no role in study design, data collection and analysis, decision to publish, or preparation of the manuscript.” iv). If any authors received a salary from any of your funders, please state which authors and which funders. | Data used in this study were collected as part of a USAID Kenya and East Africa funded program AID-615-A-17-00004, awarded to Jhpiego. The role of the funding agency was restricted to providing oversight to program implementation and efficient use of resources. While some of the authors are employees of USAID which is the funding agency, the authors did not have a role in influencing the study design, development of tools, data collection or analysis. The time spent in development of the manuscript was paid by USAID and other author affiliate organizations (APHRC and University of Nairobi), and not by the awardee, Jhpiego. |
| 3 | Please ensure that the funders and grant numbers match between the Financial Disclosure field and the Funding Information tab in your submission form. Note that the funders must be provided in the same order in both places as well. | Data used in this study were collected as part of a USAID Kenya and East Africa funded program AID-615-A-17-00004, awarded to Jhpiego. |
| 4 | Please update the completed 'Competing Interests' statement. If you have no competing interests to declare, please state “The authors have declared that no competing interests exist”. | This section has been updated accordingly to reflect *“The authors have declared that no competing interests exist”.* |
| 5 | Please ensure that you refer to Table 2 in your text as, if accepted, production will need this reference to link the reader to the Table. | Page 2 is reference in the text on page 12 of the revised manuscript |
| 6 | Please review your reference list to ensure that it is complete and correct. If you have cited papers that have been retracted, please include the rationale for doing so in the manuscript text or remove these references and replace them with relevant current references. Any changes to the reference list should be mentioned in the rebuttal letter that accompanies your revised manuscript. If you need to cite a retracted article, indicate the article’s retracted status in the References list and also include a citation and full reference for the retraction notice. | We have reviewed the reference list and ensured that it is complete and correct |
|  | **Reviewer Number 1 comments** | |
| 1 | Since the contraceptive prevalence rate is at 68%, how much further increase authors expect by the policy recommendations mentioned in this research. I would suggest that authors should highlight and find the early use of FP method after the childbirth. The early use is critical otherwise there are higher chances of unintended pregnancy or even intended pregnancy resulting in birth with shorter intervals. Such consequences have detrimental effects on maternal and child health. Therefore, I suggest using the information on the gap between childbirth and the first family planning method in the logistic regression analysis. | This requires a variable that shows “time taken” between point of delivery and use of FP. If this was available, the variable “time taken” between point of delivery and use of FP would have helped to determine factors that delay use of FP. A study in Uganda found that women who delivered at a government health facility had a shorter time to adoption of modern contraception compared with births at home [33] - this has been explained on page 16 of the manuscript |
| 2 | In the method section, authors should illustrate (from MCH & FP services guidelines of the government documents) what kind of issues get covered in the FP discussion with women. | This is covered in the introduction section on page 4 . |
| 3 | Table 1, Authors should show chi-square with p-values so that these frequency distributions would not be misled as random. | P values were not included due to Journal guidelines |
| 4 | Editorial suggestion:  4. Abstract says women having child within 0-59 months where following write-up says 0-23 months. It is creating confusion, so make it coherent throughout the article | This has been corrected to read 0-23 months and ensured consistency in the article. |
| 5 | Similarly, Well Child visit and Well-baby clinic visit are used interchangeably, again make the language consistent | This has been corrected and retained well child visits and well child clinics. |
|  | **Reviewer no. 2 comments** | |
| 1) | Introduction section:  Although you have cited the WHO recommendation for birth intervals of between 2 and 3 years. On other hand it was mentioned with literature support that “the majority (91%) of postpartum women in low- and middle-income countries (LMIC) desire to have a space birth by at least a year [11], contraceptive uptake continues to be low in this region [12, 13]. Suggestion: it will be good to add critical discussion here the reason for the desire to space birth by at least a year. | Additional information provided on desire to space births by up to a year (page 3)  Recent studies have established some of the reasons women desire to space births by at least a year. A qualitative study carried out in Malawi among post-partum women who had experienced a poor obstetric outcome indicated that women who lost a child after delivery did not wait long before conceiving, while those with normal deliveries desired to delay conception so that their bodies could recuperate, others delayed conception due their bad experiences in their last pregnancy i.e. having had cesarean or high blood pressure (1), yet another study in Uganda established that women had a clear understanding of the benefits of adequate child spacing, like allowing their bodies to recover and children getting proper nutrition and time to grow before another pregnancy, but this knowledge was difficult to translate into practice as women are disempowered to exercise child spacing. Women who used child spacing without their husband’s consent risked losing financial and social assets (2). |
| 2 | Methodology: under the research methods, it will be  important to mention the method of selecting study participants. How the setting has been selected and why? | This is explained on page 6. The study respondents were selected from the 32 wards in the targeted sub counties. A total of 768 women from 32 wards in targeted sub-counties were identified. Each of the wards was allocated 24 data points for household interviews. In order to select the number of villages per ward and the number of household per village, a list of villages was drawn from each of the wards and probability proportional to size (PPS) sampling was used to allocate the data points to the villages, while a skip pattern was applied to identify households for interview in the villages, however if the selected the household did not have an eligible mother-child (under five) pair the interviewer moved to the next household for the interview. This approach ensured equal representation of households selected for interview among the villages |
| 3 | The study findings are expected to inform the design of effective  On page 4, it was mentioned that the “strategies aimed at increasing contraceptive use and further increase the CPR and make recommendations on effective strategies for increasing contraceptive use among postpartum”. Suggestion: Make sure the study aim marries well with the purpose of your study. | The two sentences have been revised to read as follows:  The purpose of this study was to examine the factors that determine contraceptive use among postpartum women (0-23 months) in Kitui County, Kenya. The study findings are expected to inform design of interventions aimed at increasing contraceptive use among postpartum women 0-23 months. |
| 4 | Discussion:  It was discussed that “the likelihood of contraceptive use increased with the increase in the number of FP methods known. For example, women who knew 7-9 FP methods were 2.78 times more likely to use contraceptives than those who knew 0 to 3 FP methods.  Suggestion: it will add more value to this paper if authors may specify the variables that are relevant to the knowledge regarding FP and then bring in the local knowledge about why some of the participants still don’t acquire FP knowledge? | Additional information is now provided on page 17. ‘Additionally, knowledge of the various contraceptive methods is influenced by factors such as media exposure [34], place of residence, modern contraceptive ever use, place of delivery, and FP counseling during PNC [35]. |
| 5 | Regarding the discussion on “women who received FP information/ services during well-child visits had elevated odds of contraceptive use (OR = 1.51, 95%CI: 1.07, 2.13)”.  Suggestion: please make it more explicit and unfold about elevated odds of contraceptive use. | The sentence reworded to read; In this study, we found that women who received FP information/ services during well-child visits were 1.5 times more likely to use contraception when compared to Women who did not receive FP information/services during well-child visits. |
